# Influences of thermal stress on the growth biometrics, stress indicators, oxidative stress biomarkers, and histopathological alterations in European seabass, *Dicentrarchus labrax*, juveniles

**DOI:** 10.1007/s10695-025-01470-6

**Published:** 2025-03-20

**Authors:** Mohsen Abdel-Tawwab, Amira A. Omar, Riad H. Khalil, Talal A. M. Abo Selema, Salma. I. Elsamanooudy, Hend A. M. El-Saftawy, Eman A. Sabry, Reham M. Fawzy, Nashwa Abdel-Razek

**Affiliations:** 1 Department of Fish Biology and Ecology, Central Laboratory for Aquaculture Research, Agricultural Research Center, Abbassa, Abo-Hammad, Sharqia, 44662 Egypt; 2 Department of Fish Diseases and Management, Faculty of Veterinary Medicine, Kafrelsheikh University, Kafrelsheikh, Egypt; 3 Department of Poultry and Fish Diseases, Faculty of Veterinary Medicine, Alexandria University, Alexandria, Egypt; 4 Department of Physiology, Faculty of Veterinary Medicine, Cairo University, Cairo, Egypt; 5 Department of Fish Health and Management, Central Laboratory for Aquaculture Research, Agricultural Research Center, Sakha Aquaculture Research Unit, Kafrelsheikh, Egypt; 6 Department of Fish Production and Aquaculture Systems, Central Laboratory for Aquaculture Research, Agricultural Research Center, Abbassa, Abo-Hammad, Sharqia, 44662 Egypt; 7 Department of Fish Nutrition and Feed Technology, Central Laboratory for Aquaculture Research, Agricultural Research Center, Abbassa, Abo-Hammad, Sharqia, 44662 Egypt; 8 Department of Fish Health and Management, Central Laboratory for Aquaculture Research, Agricultural Research Center, Abbassa, Abo-Hammad, Sharqia, 44662 Egypt

**Keywords:** *Dicentrarchus labrax*, Heat stress, European seabass juveniles

## Abstract

This study examined how European seabass, *Dicentrarchus labrax*, juveniles are affected by heat stress in several ways, including growth biometrics, stress indicators, oxidative stress biomarkers, and histopathological changes. Our research aims to gain a better understanding of the impact of thermal stress on these parameters. Hence, European seabass juveniles (30–32 g) were exposed to temperatures of 20 °C, 23 °C, 26 °C, 29 °C, and 31 °C using a 28-day bioassay. It was noted that the fish showed better performance indices at 23 °C and 26 °C. However, fish reared at 20 °C showed intermediate growth, while the fish reared at 31 °C displayed poor performance with low survival rates. As the water temperature increased from 20 to 31 °C, the levels of glucose, cortisol, aspartate aminotransferase, and alanine aminotransferase in the fish blood also increased, suggesting that the fish were under stress. Furthermore, activities of superoxide dismutase (SOD) and catalase (CAT), as well as levels of malondialdehyde, increased significantly (*P* < 0.05) with the rise in the rearing temperature, particularly at 31 °C. This suggested that European seabass juveniles experienced oxidative stress. Additionally, the mRNA expression of SOD and CAT genes was upregulated at 31 °C compared to those reared at 23–26 °C. This high upregulation of both genes led to an increase in the secretion of SOD and CAT. Juveniles of European seabass raised in 31 °C for 28 days showed significant damage in the histological structure of their kidney, liver, and gills. In addition to fusion and blood congestion of secondary lamellae, the fish in this treatment (31 °C) displayed edema, epithelial lifting, and blood congestion of the gill epithelium. After 28 days, fish cultivated at 31 °C had sinusoid dilatation, hyperemia, and nuclear hypertrophy in their liver tissues. Furthermore, hyperemia, tubular necrosis, and severe glomerular congestion were observed in fish raised in water temperatures as high as 31 °C for 28 days. This study recommends farming European seabass at 23 °C and 26 °C, which were the optimum temperatures. By global warming due to climatic changes, water temperature may reach up to 31 °C or more, which will cause adverse effects on fish performance and increase the oxidative stress.

## Introduction

One important environmental component that affects aquatic species’ growth, survival, and health is the temperature of the rearing water (Mugwanya et al. 2022; Hao et al. 2024). The aquatic organisms may suffer negative consequences or maybe perish if the water temperature rises above the ideal level for their growth (Mugwanya et al. 2022). Therefore, global warming has become a major concern in the field of aquaculture, and it is important to understand the impact of water temperature on aquatic organisms’ growth, survival, and overall health. Global warming has led to an increase in the annual mean temperature in various aquatic ecosystems, which can affect the performance of many aquatic animals, including marine species (Almeida et al. 2015; Yilmaz et al. 2019). This rise in temperature can also make fish more susceptible to stressors in their environment, both abiotic and biotic factors (Alvarez-Lee et al. 2023). Therefore, it is crucial to investigate the effects of high temperatures, particularly on marine species, which are ecologically and economically important.

European seabass (*Dicentrarchus labrax* L.), an euryhaline and eurythermic species, is widely cultured in the Mediterranean region in cages or ponds (Vandeputte et al. 2019; Abdel-Tawwab et al. 2024) with world production of 243.9 thousand tons in 2020 (FAO 2022). In these cultured environments, the fish may experience significant fluctuations in water temperature, especially in shallow ponds. For this species, the ideal temperature range for its cultivation is thought to be between 22 and 24 °C (Claridge and Potter 1983) or possibly as high as 27 °C (Lanari et al. 2002). According to Yilmaz et al. (2019), European seabass juveniles can grow better at water temperatures ranged between 22 and 25 °C; meanwhile, their growth was adversely retarded at low temperatures (2–3 °C) and high temperature ranged (30–32 °C). Additionally, Russell et al. (1996) noted that in British waters, the fatal limit of water temperature for European seabass juvenile growth might reach 18 °C. Furthermore, populations from various geographic locations may have varied growth-temperature connections.

Due to climatic changes and the seasonal changes in temperature, juvenile European seabass reared in fishponds, cages, or natural water bodies may be exposed to different daily and/or seasonal water temperatures. The increase in water temperature is a result of global climate change (Huang et al. 2011), making thermal tolerance a critical factor for aquatic organisms to cope with stress and adapt to varying environmental conditions. Understanding thermal tolerance can also help us predict the impact of climate change on fish species distribution (Pinsky et al. 2019; Haesemeyer 2020). Thus, the goal of the current study was to assess how water temperature affected the hemato-biochemical composition, digestive enzymes, and growth performance of European seabass, *D. labrax*, juveniles. Histopathological alterations in different fish organs were also investigated at different water temperatures treatments.

## Materials and methods

### Experiment and system design

In Borg El-Arab, Alexandria, Egypt, a private fish farm provided the juvenile European seabass. The fish were kept in 200-L tanks for 2 weeks to get acclimated to the lab conditions before the experiment start. After the fish’s acclimation, fish (30 ± 2 g) were randomly distributed into 45 120-L fiberglass tanks (15 fish/tank) to represent five treatments with nine replicated tanks for each. The experiment began at 20 °C and increased by 3 °C every day using aquatic heaters until it reached 31 °C. A temperature sensor and heating systems were used to maintain the desired temperature, and the experiment equipment was set up in a climate-controlled room to reduce errors. The photoperiod regime during the experiment was 10 h of darkness and 14 h of light. The fish were fed on a commercial pellet (containing 42% crude protein and 18% fat produced by Skretting, Egypt) four times a day at 8:00, 11:00, 14:00, and 17:00 h until they appeared to be satiated. Each replicate tank was connected separately to a recirculating water system fitted with a biofilter and protein skimmers to ensure that required water quality parameters were maintained. Any uneaten feed and fish feces were removed daily, and about 30% of the tank’s water was exchanged with new water with appropriate salinity and temperature.

After 28 days of rearing period, the fish from three tanks were starved for 1 day prior to sampling and anesthesia with 30 mg/L of tricaine methanesulfonate (MS222; Sigma-Aldich, USA). To evaluate the indices of fish performance and feed utilization, all fish of each tank were counted and weighed in groups using the provided formulae:

$$\mathrm{Weight}\;\mathrm{gain}\;\%=\left[\mathrm{final}\;\mathrm{weight}\;\left(\mathrm g\right)\right];$$

$$\mathrm{Specific}\;\mathrm{growth}\;\mathrm{rate}\left(\mathrm{SGR};\%/\mathrm{day}\right)=\left[\mathrm{Ln}\left(\mathrm{final}\;\mathrm{weight};\mathrm g\right)-\mathrm{Ln}\left(\mathrm{initial}\;\mathrm{weight};\;\mathrm g\right)\right]/28\mathit;$$

$$\mathrm{Feed}\;\mathrm{conversion}\;\mathrm{ratio}\;\left(\mathrm{FCR}\right)=\mathrm{total}\;\mathrm{dry}\;\mathrm{feed}\;\mathrm{intake}/\mathrm{weight}\;\mathrm{gain};$$

$$\mathrm{First}\;\mathrm{survival}\;\left(\%\right)=100\left(\mathrm{fish}\;\mathrm{number}\;\mathrm{at}\;\mathrm{the}\;\mathrm{trial}\;\mathrm{end}/\mathrm{fish}\;\mathrm{number}\;\mathrm{at}\;\mathrm{the}\;\mathrm{trial}\;\mathrm{beginning}\right)$$

### Blood and tissues sampling

At each rearing period (0, 7, 14, and 28 days), fish from three tanks were used for blood and tissues sampling (nine fish/treatment) and these tanks were excluded from the experiment. For a full day before sampling, the fish were fasted and they were put for anesthesia right away using 30 mg/L of MS-222 (Aldrich-Sigma, USA). Serum samples were separated following centrifugation at 4000 × g for 15 min, and blood samples were extracted via caudal puncture after each fish’s weight was determined. In order to conduct additional biochemical analysis, the collected sera were kept at − 20 °C. Following blood collection, fish were dissected, and liver and mid-intestinal samples were stored at − 20 °C to measure digestive enzymes and oxidative stress indicators, respectively. Additionally, other tissues samples from the kidney, liver, and gills were gathered and kept in 10% neutral formalin for histological examination.

### The assessment of digestive enzymes activity

Using a Potter–Elvehjem glass/Tefon homogenizer, mid-intestine samples from various temperature groups were homogenized (1 g/9 mL) in phosphate-buffered saline (pH ~ 7.4). Samples were then centrifuged for 10 min at 5000 × g, and the supernatants were stored at − 80 °C until they were needed again. Using kits provided by Biodiagnostic co. (Giza, Egypt), the activities of α-amylase, lipase, and proteases were examined in accordance with the manufacturer’s instructions, following the procedures shown by Ross et al. (2000), Shihabi and Bishop (1971), and Bernfeld (1955), respectively.

### Stress indices assays

As directed by the manufacturer, the diagnostic reagent kits (Biodiagnostic Co., Giza, Egypt) were used to identify the stress biomarkers. Trinder’s (1969) methods were used to measure blood glucose levels. Foster and Dunn (1974) used a Fish Cortisol ELISA kit (Spectrum, Cairo, Egypt) to test cortisol levels. Reitman and Frankel (1957) colorimetric methods were used to measure the activity of aspartate (AST) and alanine (ALT) aminotransferases.

### Oxidative stress assays

Phosphate-buffered saline (pH ~ 7.4) was used to homogenize the liver samples from the various temperature groups (1 g:9 mL) using a Potter–Elvehjem glass/Tefon host. After centrifuging the samples for 10 min at 5000 × g, the supernatants were stored at − 80 °C until they were needed again. Diagnostic kits from Biodiagnostic Co., Giza, Egypt, were used to measure the activities of catalase (CAT) and superoxide dismutase (SOD) in liver tissues in accordance with Aebi (1984) and McCord and Fridovich (1969), respectively. For CAT, the decrease of H_2_O_2_ at 240 nm was measured using 10.6 mM H_2_O_2_ and potassium phosphate buffer (pH 7.8). One unit of SOD activity is comparable to the number of enzymes that block 50% of the ferricytochrome C reduction rate at 550 nm, according to the spectrophotochemical method used to assess the activity. Using the thiobarbituric acid approach, hepatic malondialdehyde (MDA) levels were measured spectroscopically at 532 nm as a marker of lipid peroxidation (Ohkawa et al. 1979).

### The mRNA extraction and real-time PCR analysis

The TRI-reagent (Sigma-Aldrich, Saint Louis, MO, USA) was used to extract the total mRNA (ng/µL) from the liver tissues (about 50 mg/sample) using the Qiagen RNeasy Minikit extraction kit. The reverse transcriptions were carried out using an iScriptTM cDNA synthesis Kit (Bio-Rad, Hercules, CA, USA) to produce cDNA in a 20 µL reaction using 1 µL of total mRNA at a concentration of 0.5 µg/µL. In a final volume of a 20 µL reaction, which contained 10 µL of iQTM-SYBER® Green Supermix (Bio-Rad, Hercules, CA, USA), 5 µL of free-nuclease water, 3 µL of cDNA (1:10 dilution), and 1 µL of forward and reverse primer, the real-time PCR analysis was carried out using an iCycler with the optical module. *CAT* and *SOD* genes were the target genes. Table 1 lists the precise accession numbers, annealing temperatures, and primer sequences. Real-time operating conditions included a 1-min run at 95 °C, 40 cycles at 95 °C for 10 s, and 30 s at an annealing temperature (Table 1). Every experiment included a blank control that contained nuclease-free water in place of cDNA in the final volume mix, and all reactions were carried out in duplicate for every treatment. The 2^−∆∆CT^ technique (Livak and Schmittgen 2001) was used to determine the relative gene expression levels, with β-actin serving as the housekeeping gene. In relation to the transcript levels of fish at the ideal rearing temperature, the gene expression was computed.

**Table 1:** Primer sequences of the different genes analyzed and their RT-PCR conditions for European seabass, *D. labrax*

Gene name	Primer sequence (5′–3′)	Annealing temperature (°C)	Amplicon size (bp)	GenBank Accession No
*CAT*	F: 5′-TGGGACTTCTGGAGCCTGAG-3′ R: 5′-GCAAACCTCGATCGCTGAAC-3′	60	175	FJ860003.1
*SOD*	F: 5′-CATGTTGGAGACCTGGGAGA-3′	60	182	FJ860004.1
	R: 5′-TGAGCATCTTGTCCGTGATGT-3′			
*β-actin*	F: 5′- ATCGTGGGGCGCCCCAGGCACA-3′	72	79	AJ493428.1
	R: 5′-CTCCTTAATGTCACGCACGATTTC-3′			

*CAT* catalase, *SOD*, superoxide dismutase, *β-actin* beta actin (a housekeeping gene)

### Histopathological examinations

Following conventional protocols, samples from the kidneys, liver, and gills were prepared for histological examination. To prevent post-mortem alterations and shrinking, tissue samples were preserved in Bouin’s fixative for a whole day. Samples were then washed in xylene, embedded in paraffin wax, and dehydrated in a series of graded ethanol. Hematoxylin and eosin (H&E) was used to stain sections (5 μm) in accordance with the instructions of Bancroft and Gamble (2013). An attached digital camera was used to take pictures of the stained slices and to inspect them under a light microscope.

### Statistical analysis

To elucidate the effects of water temperature, rearing time, and their interaction, a two-way ANOVA was used. Levene’s test was performed to examine variance homogeneity in the dependent variables, and Kolmogorov–Smirnov was used to verify that the data was normal. Then, at a significance threshold of *P* < 0.05, Tukey’s HSD test was used to assess significant differences between treatments. All statistical analyses were done utilizing SPSS statistical software version 26 (SPSS, Richmond, VA, USA) as described in Dytham (2011).

## Results

### Growth parameters

**Fig. 1:**
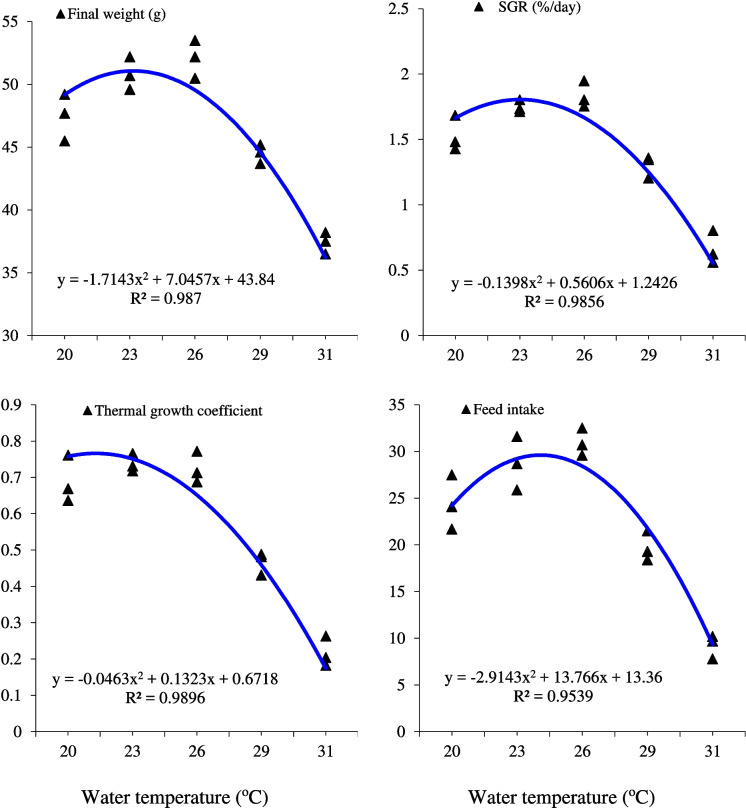
The relationship between final weight (g), specific growth rate (SGR; %/day), thermal growth coefficient, and feed intake (g feed/fish) of European sea bass, *D. labrax*, juveniles reared at different water temperatures for 28 days (*n* = 3)

It is observed that fish reared at 23 °C and 26 °C for 28 days displayed significantly (*P* < 0.05) higher final weight, weight gain percentage (WG%), specific growth rate (SGR), and thermal growth coefficient (TGC) compared to fish raised at other water temperatures (Table 2). Furthermore, fish kept at both temperatures (23 °C and 26 °C) consumed more feed (28.74 ± 1.65 and 30.93 ± 0.85 g feed/fish, respectively) than those in other treatments, with no significant (*P* > 0.05) differences in feed conversion ratio (FCR) values (1.46–1.48) among treatments (Table 2). Overall, all growth biometrics and feed utilization parameters were significantly (*P* < 0.05) the lowest in fish reared at 31 °C. The lowest fish survival was observed in fish raised at 31 °C for 28 days (83.3 ± 3.33%; Table 2). The fitting curves (Fig. 1) depicting the relationships between final weight (g), SGR (%/day), thermal growth coefficient, and feed intake (g feed/fish) of European seabass juveniles reared at different water temperatures for 28 days indicate that the optimal rearing temperature for this fish species is 24.5 °C (Fig. 1).

**Table 2:** Growth performance of European seabass, *D. labrax*, juveniles exposed to different water temperatures (℃) for 28 days (*n* = 3)

Temperature (℃)	Initial weight (g)	Final weight (g)	Weight gain (%)	SGR (%/day)	TGC	Feed intake (g feed/fish)	FCR	Survival (%)
20	30.9 ± 0.31	47.5 ± 1.07 b	53.7 ± 3.38 b	1.54 ± 0.078 b	0.52 ± 0.037 a	24.45 ± 1.68 b	1.48 ± 0.077	96.7 ± 3.33 a
23	31.1 ± 0.23	50.8 ± 0.75 a	63.3 ± 1.26 a	1.75 ± 0.074 a	0.60 ± 0.014 a	28.74 ± 1.65 a	1.46 ± 0.081	96.7 ± 3.33 a
26	31.2 ± 0.19	52.1 ± 0.87 a	67.0 ± 2.75 a	1.83 ± 0.058 a	0.64 ± 0.025 a	30.93 ± 0.85 a	1.48 ± 0.191	100.0 ± 0.00 a
29	30.9 ± 0.17	44.5 ± 0.44 c	44.0 ± 1.99 c	1.30 ± 0.50 c	0.44 ± 0.018 b	19.72 ± 0.92 c	1.45 ± 0.130	90.0 ± 5.77 ab
31	31.1 ± 0.30	37.4 ± 0.49 d	20.3 ± 2.48 d	0.66 ± 0.073 d	0.22 ± 0.024 c	9.25 ± 0.73 d	1.47 ± 0.127	83.3 ± 3.33 b
*P* value	0.912	< 0.0001	< 0.0001	< 0.0001	< 0.0001	< 0.0001	0.893	0.026

*SGR* specific growth rate, *TGC* thermal growth coefficient, *FCR* feed conversion ratio

Means followed by different letters in the same column are significant different at *P* < 0.05

### Digestive enzyme activities

**Fig. 2:**
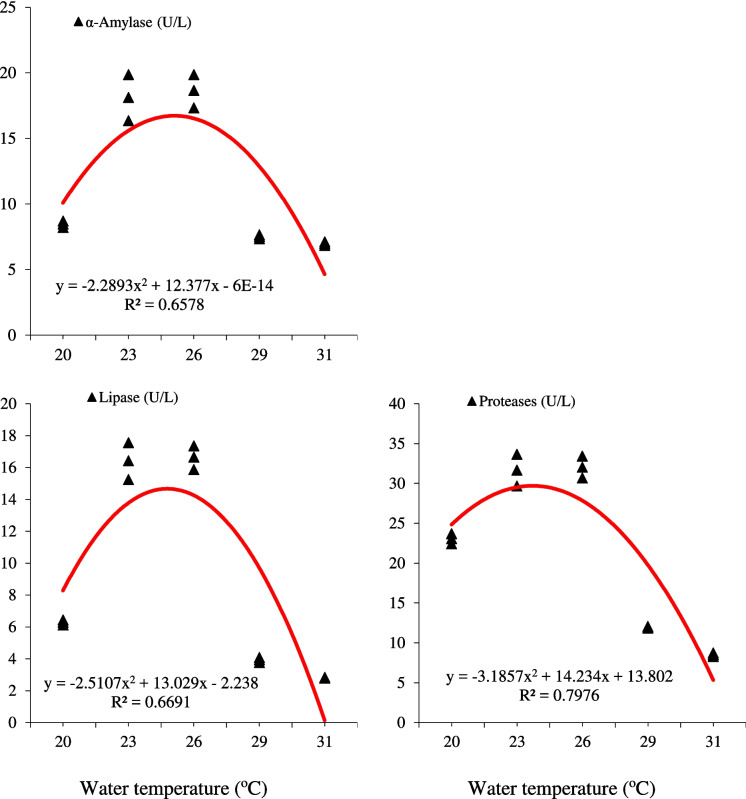
The relationship between α-amylase, proteases, and lipase activities in mid-intestine of European sea bass, *D. labrax*, juveniles reared at different water temperatures for 28 days (*n* = 3)

The water temperature, rearing period, and their combination had a substantial (*P* < 0.05) effect on the activities of mid-intestinal α-amylase, lipase, and proteases (Table 3). Up to 14 and 28 days, the aforementioned enzymes’ activity rose; nonetheless, there was no discernible (*P* > 0.05) difference between the two time points. However, their optimum activities were gradually elevated with increasing water temperature up to 23–26 °C, after which those activities declined (Table 3). The relationship between α-amylase, proteases, and lipase activities in the mid-intestine of European seabass, *D. labrax*, reared at different water temperatures for 28 days suggests that the ideal water temperature for the activities of those enzymes is 25 °C (Fig. 2).

**Table 3:** Effects of water temperature (℃) and exposure time (days) on mid-intestinal digestive enzyme activities of European seabass, *D. labrax*, juveniles

Temperature (℃)	Days	α-Amylase (U/mg protein)	Proteases (U/mg protein)	Lipase (U/mg protein)
		Individual treatment means^a^		
20	0	8.0	16.1	3.8
	7	8.2	18.9	4.4
	14	8.3	21.0	5.8
	28	8.5	23.1	6.3
23	0	8.0	16.2	3.9
	7	12.0	23.2	8.1
	14	15.8	29.9	13.2
	28	18.1	31.7	16.4
26	0	8.0	16.3	4.0
	7	12.2	23.4	8.2
	14	15.9	30.4	13.7
	28	18.6	32.1	16.6
29	0	8.1	15.9	4.1
	7	10.0	15.4	4.9
	14	9.0	13.9	4.8
	28	7.5	12.0	3.9
31	0	8.2	16.3	3.6
	7	8.2	13.9	3.5
	14	7.9	11.9	3.3
	28	7.0	8.5	2.8
Pooled SE		0.430	0.817	0.478
		Means of the main effects^b^		
20		8.2 b	19.8 b	5.1 b
23		13.5 a	25.2 a	10.4 a
26		13.7 a	25.5 a	10.6 a
29		8.7 b	14.3 c	4.4 b
31		7.8 b	12.6 c	3.3 b
	0	8.1 y	16.2 y	3.9 z
	7	10.1 xy	19.0 xy	5.8 yz
	14	11.4 x	21.4 x	8.2 xy
	28	11.9 x	21.5 x	9.2 x
Two-way ANOVA		*P* value		
Temperature		< 0.001	< 0.001	< 0.001
Days		< 0.001	< 0.001	< 0.001
Temperature × days		< 0.001	< 0.001	< 0.001

^a^Treatment means represent the average values of three tanks per treatment for each sampling time

^b^Main effect means followed by the same letter are not significantly different at *P* < 0.05 by Tukey’s HSD test; a, b, and c for water temperature and x, y, and z for sampling period

### Stress indices

It was noted that changes in blood AST, ALT, glucose, and cortisol levels activities were significantly (*P* < 0.05) affected by both rearing temperature and the interaction between temperature and rearing period, with no effects (*P* > 0.05) observed for rearing period alone (Table 4). Their highest levels of both enzymes were found in fish reared at 29 °C and 31 °C, while no changes in AST and ALT activities were seen in fish reared at 20–26 °C. Fish reared at 23–26 °C showed no changes in glucose levels, while the lowest cortisol levels were observed in fish reared at 20–26 °C (Table 4).

**Table 4:** Effects of water temperature (℃) and exposure time (days) on blood AST, ALT, glucose, and cortisol of European seabass, *D. labrax*, juveniles

Temperature (℃)	Days	AST (U/L)	ALT (U/L)	Glucose (mg/dL)	Cortisol (ng/mL)
		Individual treatment means^a^			
20	0	9.96	22.22	64.8	57.0
	7	10.18	21.37	61.8	54.4
	14	9.93	19.33	54.5	50.9
	28	9.51	19.83	51.7	48.1
23	0	9.96	22.07	60.4	58.8
	7	10.27	21.50	53.8	52.8
	14	9.93	20.98	48.4	46.7
	28	9.51	19.22	41.8	42.6
26	0	10.49	21.83	59.4	59.1
	7	10.73	20.73	50.6	52.4
	14	9.93	19.52	44.9	46.7
	28	9.45	16.70	39.9	42.3
29	0	10.34	22.57	59.9	60.3
	7	11.52	22.90	57.6	60.5
	14	12.42	24.37	64.1	65.7
	28	14.2	25.67	69.4	68.1
31	0	10.79	21.83	62.8	59.2
	7	11.99	23.63	64.9	62.9
	14	13.86	26.22	70.2	73.0
	28	15.86	28.85	78.0	80.8
Pooled SE		1.849	2.746	9.598	9.995
		Means of the main effects^b^			
20		9.90 b	20.69 b	57.9 b	52.6 c
23		9.92 b	20.94 b	51.1 c	50.2 c
26		10.15 b	19.70 b	48.7 c	50.1 c
29		12.12 a	23.88 a	62.8 b	63.6 b
31		13.13 a	25.13 a	69.0 a	69.0 a
	0	10.31	22.10	61.3	58.9
	7	10.94	22.03	57.7	56.6
	14	11.21	22.08	56.4	56.6
	28	11.71	22.05	56.1	56.4
Two-way ANOVA		*P* value			
Temperature		< 0.0001	< 0.0001	< 0.0001	< 0.0001
Days		0.109	0.947	0.194	0.538
Temperature × days		< 0.0001	< 0.0001	< 0.0001	< 0.0001

^a^Treatment means represent the average values of three tanks per treatment for each sampling time

^b^Main effect means followed by the same letter are not significantly different at *P* < 0.05 by Tukey’s HSD test; a, b, and c for water temperature and x, y, and z for sampling period

### Oxidative stress biomarkers

It is noticeable that the changes in hepatic SOD and CAT activities, as well as MDA levels, are significantly (*P* < 0.05) affected by the rearing temperature, rearing period, and their interaction (Table 5). The highest levels of SOD and CAT were found in fish reared at 31 °C, whereas the highest MDA levels were found in fish reared at 29 °C and 31 °C with no significant (*P* > 0.05) differences between the two treatments. Fish reared at 20–26 °C showed no marked changes (*P* > 0.05) in oxidative stress indices (Table 5). On the other hand, the lowest hepatic SOD and MDA levels were detected in fish reared for 14 days and 28 days, but the lowest CAT activity was observed in fish reared for 28 days (Table 5).

**Table 5:** Effects of water temperature (℃) and exposure time (days) on hepatic oxidative stress indices of European seabass, *D. labrax*, juveniles

Temperature (℃)	Days	SOD (U/mg protein)	CAT (U/mg protein)	MDA (nmol/mg protein)
		Individual treatment means^a^		
20	0	9.4	8.1	1.55
	7	8.9	7.4	1.46
	14	8.7	6.9	1.30
	28	6.9	6.2	1.19
23	0	10.3	8.8	1.61
	7	9.5	7.4	1.40
	14	8.9	6.9	1.30
	28	8.7	6.3	1.19
26	0	10.7	9.9	1.52
	7	10.0	8.3	1.34
	14	9.3	7.6	1.21
	28	8.9	6.1	1.12
29	0	12.0	13.9	1.59
	7	10.9	11.0	1.54
	14	9.9	8.4	1.78
	28	9.3	6.9	1.96
31	0	16.2	20.2	1.37
	7	13.1	17.3	1.52
	14	11.8	11.7	2.02
	28	8.6	7.2	2.31
Pooled SE		1.98	4.19	0.305
		Means of the main effects^b^		
20		8.5 c	7.2 c	1.38 b
23		9.4 c	7.4 c	1.38 b
26		9.7 c	8.0 c	1.30 b
29		10.5 b	10.1 b	1.72 a
31		12.4 a	14.1 a	1.81 a
	0	11.7 x	12.2 x	1.53 x
	7	10.5 x	10.3 y	1.45 y
	14	9.7 xy	8.3 z	1.52 x
	28	8.5 y	6.5 L	1.55 x
Two-way ANOVA		*P* value		
Temperature		< 0.001	< 0.001	< 0.001
Days		< 0.001	< 0.001	< 0.001
Temperature × days		< 0.001	< 0.001	< 0.001

^a^Treatment means represent the average values of three tanks per treatment for each sampling time

^b^Main effect means followed by the same letter are not significantly different at *P* < 0.05 by Tukey’s HSD test; a, b, and c for water temperature and x, y, z, and L for sampling period

**Fig. 3:**
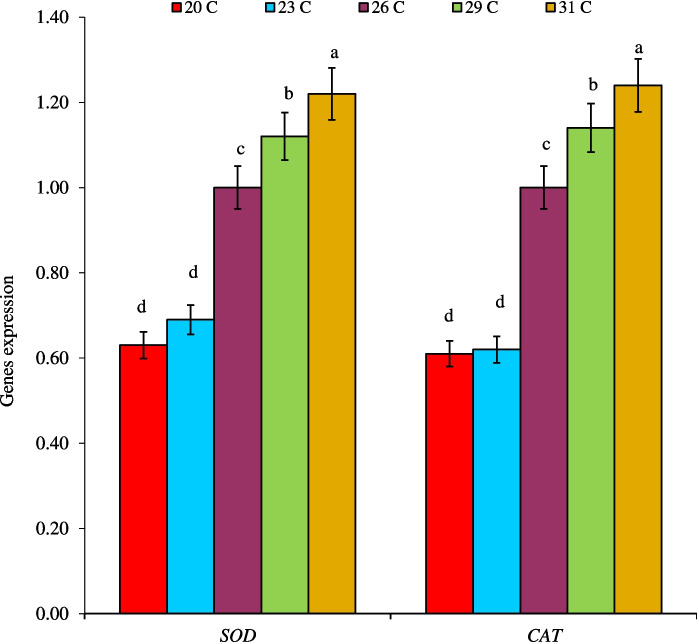
The mRNA expression levels of *SOD* and *CAT* genes in liver tissues of European sea bass, *D. labrax*, juveniles reared at different water temperatures (℃) for 28 days*.* Bars assigned by different letters are significantly differed at *P* < 0.05 (*n* = 3 for each sampling time)

The mRNA expression of *SOD* and *CAT* genes was significantly (*P* < 0.05) upregulated as the rearing temperature increased, with the highest expression detected in fish reared at 31 °C, followed by those in fish reared at 29 °C (Fig. 3). The lowest mRNA expression of both genes was found in fish reared at 20 °C and 23 °C with no significant (*P* > 0.05) differences between them.

### Histopathological changes in gill tissues

**Fig. 4:**
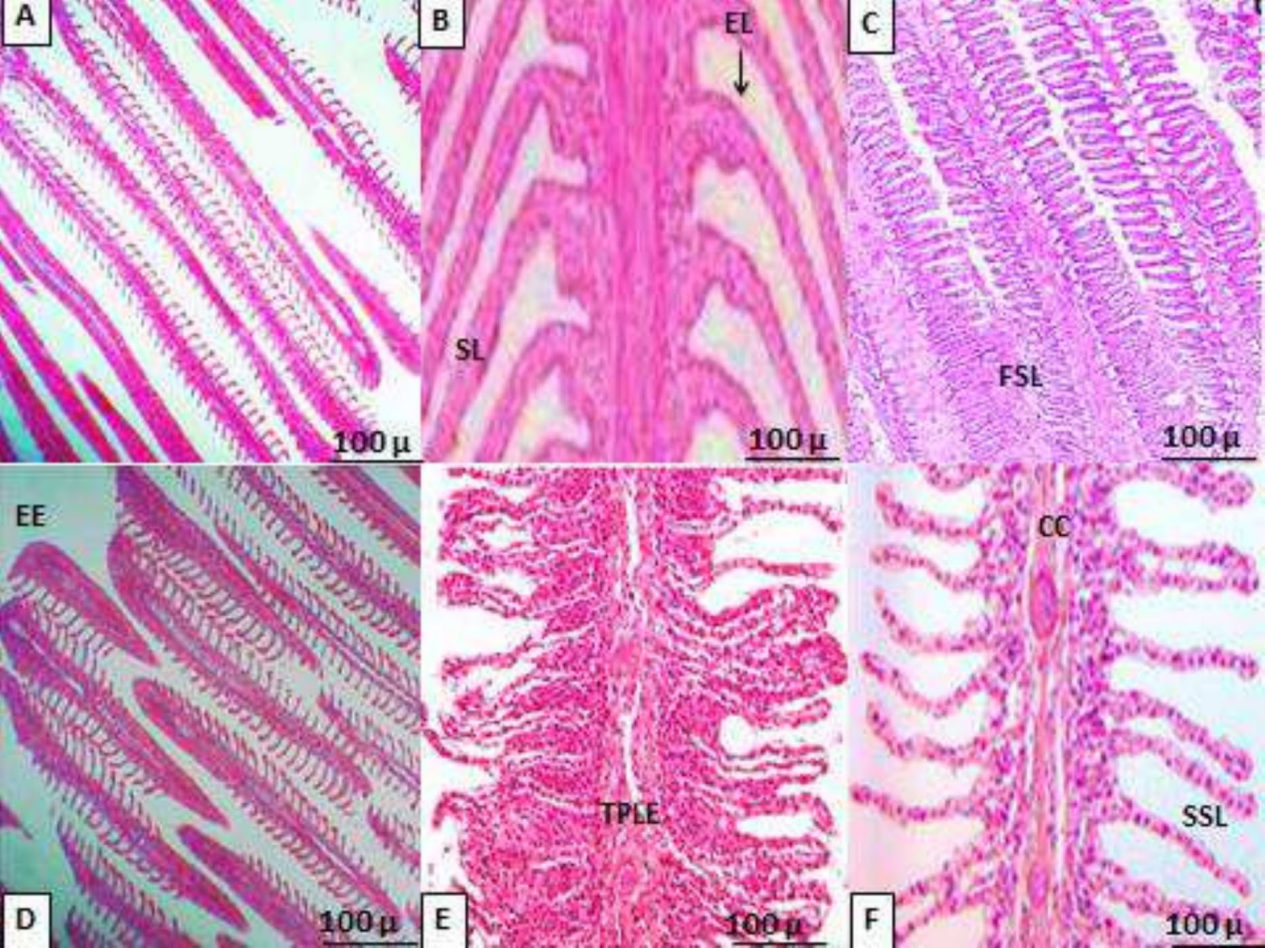
**A–F** Histopathological evaluation of gills tissues of European seabass, *D. labrax*, juveniles reared at different water temperatures for 28 days. **A** The control fish before beginning of the experiment; × 10; **B** fish exposed to 20 °C; × 10; **C** fish exposed to 23 °C; × 20; **D** fish exposed to 26 °C; × 20; **E** fish exposed to 29 °C; × 20; **F** fish exposed to 31 °C; × 20.EL, epithelial lifting; SL, secondary lamellae; EE, edema of epithelium; CC, blood congestion; CSL, curling of secondary lamellae; FSL, fusion of the secondary lamella; TPLE, thickening of primary lamellae epithelium; SSL, shortening of secondary lamellae

After 28 days of exposure to varying temperatures, gills tissues of European seabass were histologically analyzed and compared to fish from the 0 day (Fig. 4A–F). Double rows of filaments or primary lamellae aligned perpendicular to the secondary lamellae were visible in gills tissues of the control fish. The thick stratified epithelium that lined the principal gills lamellae between the secondary lamellae was known to include a large number of cells that secreted mucus and cells that were enriched in chloride. Figure 4A shows that the secondary gills lamellae were encircled by a thin layer of epithelial cells and were extensively vascularized. The histopathological examination of the gills tissue at 20–26 °C after 28 days showed very little variation, summarized as slight curling and fusion of secondary lamellae. Furthermore, the principal lamellae thickened and shortened, and there was edema and mild lifting of the epithelium (Fig. 4B–D). On the other hand, gills morphology was disturbed at 29 °C and 31 °C, exhibiting edema of the gills epithelium, blood congestion, and epithelial lifting (Fig. 4E–F). Blood congestion and secondary lamellar fusion were observed in the histology of the gills tissues of fish raised for 28 days at 31 °C (Fig. 4F). The epithelium was seen to be lifted and edematous, and the primary lamellae were thickened and shortened, while the secondary lamellae showed significant curling and fusing (Fig. 4F).

### Histopathological changes in liver tissues

**Fig. 5:**
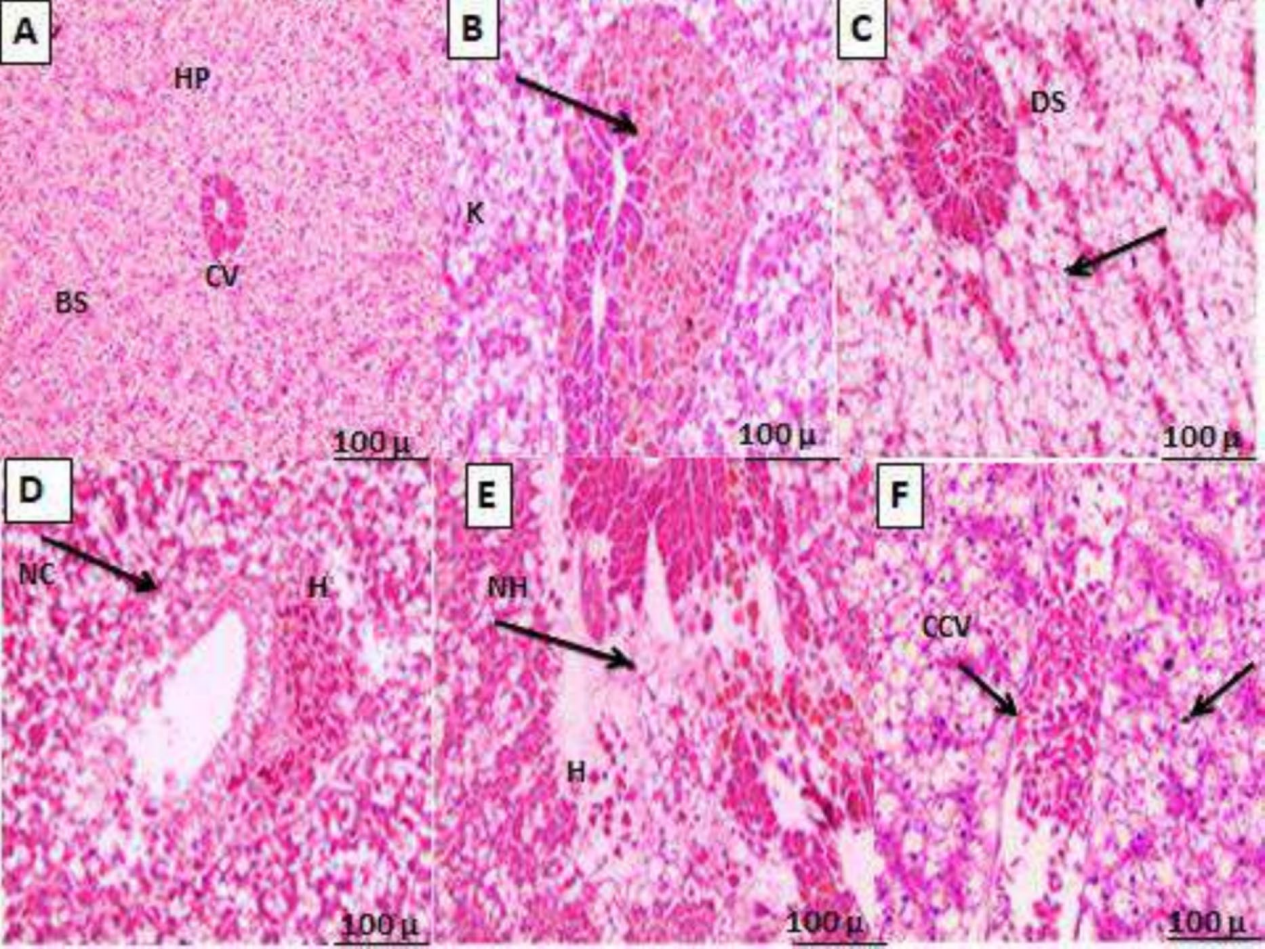
**A–F** Histopathological evaluation of liver’s tissues of European seabass, *D. labrax*, juveniles reared at different water temperatures for 28 days. **A** The control fish before beginning of the experiment; × 10; **B** fish exposed to 20 °C; × 10; **C** fish exposed to 23 °C; × 20; **D** fish exposed to 26 °C; × 20; **E** fish exposed to 29 °C; × 20; **F** fish exposed to 31 °C; × 20. HP, hepatocytes; BS, blood sinusoid; CV, central vein; K, karyolysis; DS, dilatation of sinusoids; NC, necrosis; NH, nuclear hypertrophy; CCV, congestion of central vein; H, hyperemia

After being exposed to varying water temperatures for 28 days, liver tissues of European seabass were histologically analyzed and contrasted with those of the 0 day of the exposure (Fig. 5A–F). Prior to the trial, the liver tissues of the control (0 day) group displayed normal sinusoids and hepatocytes. Hepatic cell cords are structures that resemble cords and are generated by these hepatocytes (Fig. 5A). In fish reared at 20–26 °C for 28 days, there was a slight dilation of sinusoids and moderate nuclear hypertrophy (Fig. 5B–D). On the other hand, fish raised at 29 °C and 31 °C showed altered liver morphology (Fig. 5E–F). After 28 days, the severity of these histological changes increased in fish cultivated at 29 °C, resulting in nuclear hypertrophy, necrosis, central vein injury, hyperemia, and sinusoidal dilatation (Fig. 5E). Lastly, nuclear hypertrophy, hyperemia, and sinusoid dilatation were particularly noticeable at 31 °C following 28 days of heat exposure (Fig. 5F).

### Histopathological changes in kidneys tissues

**Fig. 6:**
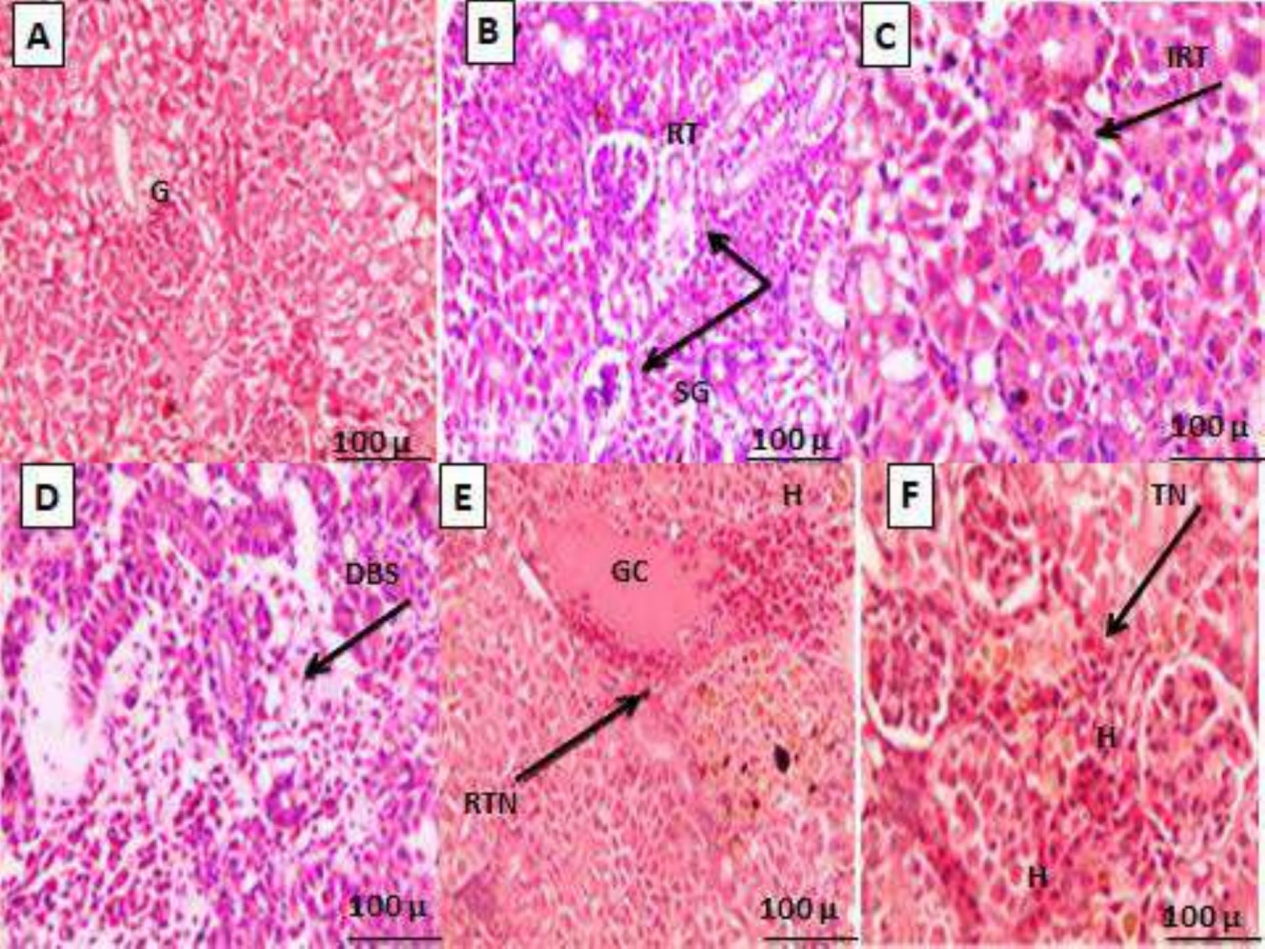
**A–E** Histopathological evaluation of kidney’s tissues of European seabass, *D. labrax*, juveniles reared at different water temperatures for 28 days. **A** The control fish before beginning of the experiment; × 10; **B** fish exposed to 20 °C; × 10; **C** fish exposed to 23 °C; × 20; **D** fish exposed to 26 °C; × 20; **E** fish exposed to 29 °C; × 20; **F** fish exposed to 31 °C; × 20.G, glomerulus; RT, renal tubules; IRT, increased diameter of renal tubules; DBS, dilation of Bowman’s space; SG, shrinkage of glomerulus; RTN, renal tubules necrosis; H, hyperemia; TN, tubular necrosis; GC, glomerular congestion

Histological analysis of European seabass's kidney tissues following exposure to varying water temperatures was performed, and the results were compared to those of the 0 day of exposure (Fig. 6A–F). Nephrons, which are normally functioning kidney units made up of well-organized renal corpuscles and renal tubules, are seen in the kidney tissues of the control fish. There are hemopoietic tissues all around these structures. It is not surprising that the renal corpuscles have an approximately spherical shape (Fig. 6A). Histopathological analysis of the kidney tissues of fish raised for 28 days at 20 °C, 23 °C, and 26 °C reveals a small dilatation of Bowman’s space and an increase in the renal tubules’ diameter (Fig. 6B–D). However, disrupted kidney tissue morphology is observed in fish reared at 29 °C and 31 °C (Fig. 6E–F). After 28 days, the histological changes showed hyperemia, glomerular shrinkage, renal tubular necrosis, and a considerably dilated Bowman’s gap. They also increased dramatically at 29 °C. Similarly, tubular necrosis, hyperemia, and widespread glomerular congestion are seen at 31 °C after 28 days (Fig. 6F). These results imply that renal tissue shape in European seabass juveniles is negatively impacted by high water temperatures.

## Discussion

### Growth biometrics

In an era of striking climate changes, increased marine water temperature severely affects the performance and health of aquatic animals. Fluctuations in environmental temperatures influence feed intake, nutrient digestion, assimilation, and nutrient absorption (Abdel-Tawwab and Wafeek 2014; Fu et al. 2018; Volkoff and Rønnestad 2020; Islam et al. 2022). In the current study, fish reared at 23–26 °C produced the optimum growth biometrics, while those stressed by high water temperatures (31 °C) exhibited retarded growth indices. These results could be attributed to the better feed consumption by fish reared at moderate water temperature (23–26 °C) compared to other temperature treatments. According to Claridge and Potter (1983), the optimum temperature for cultivating this fish species is between 22 and 24 °C, or even 27 °C. Lanari et al. (2002) reared European seabass in water temperatures ranged from 13 to 31 °C and they reported that fish performance began to rise at 22 °C and continued up to 27 °C. In a previous study, Yilmaz et al. (2019) raised young European seabass (*D. labrax*) at three different temperatures of 15 °C, 20 °C, and 25 °C for 60 days and they found that the optimum fish growth was observed at 25 °C. Zhou et al. (2021) reared Juvenile European seabass at 10, 15, and 20 °C for 60 days and they observed that growth indices in both 10 and 15 °C were all significantly lower than the fish reared at 20 °C.

The retarded fish growth at the high rearing temperature (31 °C) could be linked with low feed consumption in this treatment. This is because fish under stress due to their exposure to high temperatures divert their growth-related energy toward repairing injured tissues, which inhibits their ability to grow (Islam et al. 2022). Prior researches have been demonstrated that feed intake, feed conversion ratio, and specific growth rate are adversely affected by severe temperatures that exceed the ideal thermal range for specific aquaculture species (Volkoff and Rønnestad 2020; Islam et al. 2022). Hur et al. (2008) noted that when olive flounder (*Paralichthys olivaceus*) were exposed to water temperatures higher than 25 °C, their feed intake, body length, and body weight decreased. Bull trout (*Salvelinus confluentus*) raised at ≥ 22 °C showed a decrease in feed utilization, according to Selong and McMahon (2001). Additionally, Ilham and Fotedar (2016) found that yellowtail kingfish (*Seriola lalandi*) exposed to 26 °C performed worse in terms of growth. According to a recent study by Ignatz et al. (2020), raising female triploid Atlantic salmon (*Salmo salar*) at high temperatures (16 °C) results in large financial losses because of the high feed intake, low feed utilization rates, and slower growth rates as measured by the thermal-unit growth coefficient. Zhao et al. (2024) reared stream groupers, *Acrossocheilus fasciatus*, fry (0.57 ± 0.16 g) at six different temperatures of 12 °C, 16 °C, 20 °C, 24 °C, 26 °C, and 28 °C for a period of 60 days. They found that all growth and feeding indices were higher in the 26 °C group than other groups.

The survival rates were the lowest in fish reared at 31 °C (83.3%), while the survival rate of fish reared at 20–28 °C was 96.7–100% (Table 2). Exposing fish to high water temperatures can alter the function of the stress axis and the response to further stressors, possibly compromising the long-term coping capacity of the animal (Samaras et al., 2018). Therefore, it is speculated that this may be the reason for the lowest survival rate in group 31 °C. Similar results were detected by Abdel-Tawwab and Wafeek (2014) who noticed low survivability of Nile tilapia, *Oreochromis niloticus*, fingerlings (26.1 g) reared at 32 °C (90%) compared with other fish groups reared at 20, 24, and 28 °C (93.3–100%). Zhao et al. (2024) found also that the survival of stream groupers fry was the lowest at 28 °C group (79.67%), while the other fish groups were more than 95%.

### Digestive enzyme activities

The optimal activities of mid-intestinal digestive enzymes (α-amylase, proteases, and lipase) were observed in fish reared at temperatures between 23 and 26 °C. Conversely, below and above those water temperatures, the activities of digestive enzymes were decreased. The concentration and activity of digestive enzymes in the fish gut are eventually impacted by high temperatures, which are known to reduce feed intake and, as a result, lower nutrient retention for aquaculture species (Mugwanya et al. 2022). According to Solovyev and Izvekova (2016), elevated temperatures can also alter the pH and ion concentrations in the gut, which can inhibit the activity of digestive enzymes. It should be noted that enzymes work optimally within specific temperature ranges and their activity can be hindered if the temperature falls above or below these ranges. At temperatures above their optimal range, enzyme-catalyzed reactions can be slowed or stopped due to enzyme denaturation, while at temperatures below the critical minimum, enzymes can become inactivated (Mazumder et al. 2018). For example, Zhao et al. (2009) found that catfish raised at 32 °C had lower trypsin, pepsin, and lipase activity than those raised at 24 °C and 28 °C. Similar findings were made by Ahmad et al. (2014), who examined how different temperatures affected feed consumption and digestive enzyme activity in catfish, *Clarias batrachus*. They discovered that fish raised at 30 °C and 35 °C had significantly lower levels of proteases, trypsin, and chymotrypsin activity, suggesting a metabolic shift under extreme heat stress. Additionally, raising turbot (*Scophthalmus maximus* L.) juveniles at higher temperatures (20 °C) inhibited the activity of lipases, amylase, and total alkaline protease, according to Guerreiro et al. (2016). Jiang et al. (2021) recently discovered a similar pattern in rainbow trout (*Oncorhynchus mykiss*), where trypsin, lipase, and amylase activity decreased when the fish were raised at temperatures higher than their thermal threshold (21 °C). Furthermore, Chen et al. (2022) found that common carp (*Cyprinus carpio*) raised in high-temperature stress (30 °C) had lower activity of the digestive enzymes, lipase and α-amylase.

### Stress indices

Blood indicators are frequently used to evaluate fish health in general. Because glucose molecules are important for animal bioenergetics, which results in ATP generation, blood glucose, in particular, is sensitive to temperature fluctuations in water (Lucas 1996). Additionally, glucose levels are closely related to cortisol levels, making them commonly used as stress indicators (Wendelaar Bonga 1997; Barton 2002; Martínez-Porchas et al. 2009). Our results in the present study show that the blood glucose and cortisol levels increased significantly in fish exposed to 31 °C, compared to those reared at 23–26 °C. This increase is due to the physiological responses of cortisol on chromaffin cells, resulting in glycogenolysis, gluconeogenesis, and modulating cardiovascular and respiratory function (Reid et al. 1992, 1998). This finding is consistent with the study of Khieokhajonkhet et al. (2023) who observed a linear increase in glucose and cortisol levels in goldfish (*Carassius auratus*) exposed to increased temperatures, with the highest levels observed at 34 °C.

The levels of ALT and AST in the serum can serve as indicators of liver damage (Abdel-Tawwab and Wafeek 2017; Yu et al. 2019). The liver is the most sensitive organ to environmental changes and is an essential component of metabolism. Assessing its health state under various circumstances is essential. These enzymes’ increased activity with extended exposure to heat stress may be a sign of organ failure or tissue injury (Abdel-Tawwab and Wafeek 2017). In our study herein, European seabass juveniles reared at 31 °C showed higher levels of AST and ALT compared to those reared at 23 °C and 26 °C. This increase has also been reported in Wuchang bream, *Megalovrama amblycephala* (Ming et al. 2012), tilapia (Abdel-Tawwab and Wafeek 2017; Bao et al. 2018; Zeng et al. 2021), catfish (Dalvi et al. 2017), Atlantic cod (Larsen et al. 2018), and olive flounder (Lim et al. 2021) under heat stress. Similarly, Khieokhajonkhet et al. (2023) found a significant increase in AST and ALT levels in goldfish exposed to increasing temperatures for 10 weeks (27 °C, 30 °C, and 34 °C). In another study by Hao et al. (2024), heat stress was found to increase AST and ALT activities in juvenile greater amberjack (*Seriola dumerili*), indicating an increase in the consumption of free amino acids for energy production (Yang et al. 2020).

### Oxidative stress biomarkers

Reactive oxygen species (ROS) are produced as a result of irregular oxidative stress, which is brought on by both biotic and abiotic stress. This frequently results in more severe outcomes, including the damage of important macromolecules like DNA, lipid peroxidation, and protein carboxylation (Meng et al. 2017; Murphy et al. 2019). Numerous antioxidant enzymes, including SOD and CAT, are found in aquatic creatures and are essential for preventing oxidative damage and preserving the normal physiological state of cells by removing ROS (Meng et al. 2017; Murphy et al. 2019; Mugwanya et al. 2022). MDA is a marker of ROS-induced cellular membrane damage, and lipid peroxidation leads to its buildup (Murphy et al. 2019; Mugwanya et al. 2022). Fish tissues that accumulate MDA frequently experience homeostasis impairment, which reduces the organism’s capacity for adaptation and stress tolerance (Jia et al. 2020). In the current study, heat stress caused oxidative stress in European seabass, and the fish responded by enhancing the activity of antioxidant enzymes and upregulating the expression of antioxidant-related genes. As a result, SOD and CAT activities increased as well as MDA levels increased as water temperatures rose to 31 °C. The mRNA expression of *SOD* and *CAT* genes was also upregulated at 31 °C compared to those reared at 23–26 °C. This high upregulation of both genes led to the secretion of high levels of SOD and CAT enzymes. The increased activity of SOD and CAT, along with MDA increases, has been observed in several aquatic organisms under heat stress (Meng et al. 2017; Liu et al. 2019; Murphy et al. 2019; Mugwanya et al. 2022). Previous studies on goldfish, *C. auratus* (Lushchak and Bagnyukova 2006), medaka, *Oryzis melastigma* (Almeida et al. 2015), olive flounder (Lu et al. 2016), seabream, *Sparus aurata* (Madeira et al. 2016), longtail southern cod, *Patagonotothen ramsayi* (Forgati et al. 2017), hybrid tilapia (Musa et al. 2021), and juvenile greater amberjack (Hao et al. 2024), have shown that heat stress significantly increased SOD and CAT activities, coupled with high MDA levels. According to Vinagre et al. (2012) and Madeira et al. (2013), European seabass, *D. labrax* and white seabream, *Diplodus sargus*, respectively, exposed to both low temperatures (12–18 °C) and high temperatures (28 °C, 32 °C, and 33.3 °C) for 14 days showed higher levels of glutathione-S-transferase, CAT, and MDA compared to those reared at 24 °C. For the same fish species, significant increases in the antioxidant indices were observed at 8 °C and > 32 °C compared to those reared at 16 °C (Islam et al. 2020, 2022).

### Histological examinations

When fish are under environmental stress, they can undergo internal anatomical changes that are detrimental or adaptive. If these alterations are not corrected or compensated for, the body weakens, losing its ability to cope with other stressors. Because the gills come into direct contact with the outside world, they are especially vulnerable to high water temperatures (Shin et al. 2018). Fish raised at 31 °C for 28 days in the current study displayed gills epithelium edema, blood congestion, and epithelial lifting. In addition, the primary lamellae thickened and shrank, and there was blood congestion and secondary lamellar fusion. Many studies have found that heat stress can cause oxidative damage, leading to structural damage of fish gills (Chen et al. 2021). For example, Mozampique tilapia (*Oreochromis mossambicus*) exposed to heat stress at 40 °C displayed epithelial cell swelling (Hocutt and Tilney 1985). Similarly, Japanese flounder exposed to 32 °C showed severe damage to their gills, including epithelial lifting, hyperplasia, and hyperemia (Liu et al. 2014). Chen et al. (2021) observed that exposing pikeperch (*Sander lucioperca*) to heat stress at 29 °C resulted in direct damage to the structure of their gill tissue, with the degree of damage increasing as the heat stress duration prolonged. According to Chen et al. (2023), the substantial fusion of the gill lamellae seen in this study would directly decrease the respiratory surface area, which might lessen the harm that stressors do to the gills interior. Nevertheless, this decrease in respiratory surface area could result in issues like hypoxia and respiratory failure.

The main organ in charge of detoxification, the liver, is extremely susceptible to harm. Liver tissue alterations may provide as early warning signs of rising temperatures (Hall et al. 2000). In the present study, juvenile European seabass exposed to water at 31 °C for 28 days had nuclear hypertrophy, necrosis, central vein injury, and sinusoidal dilatation in addition to hyperemia. Specifically, in the liver, there was a considerable increase in the number of melanomacrophage centers, bile duct hyperplasia, and vacuolar degeneration. This suggests that the high temperature caused liver damage, which is supported by the increased activities of AST and ALT. It is well known that damaged cells and organelles release both enzymes, demonstrating the harmful effects of high temperatures on the liver tissues.

Goldfish cultivated at 30 °C exhibited distributed cytoplasmic vacuoles on expanding hepatocytes, as well as anomalies in the major hepatic vein, nuclei displacement, partially hepatic hypertrophy, and infiltration of inflammatory cells, according to a related study by Khieokhajonkhet et al. (2023). According to Khieokhajonkhet et al. (2023), fish raised at 34 °C showed more severe hepatic hypertrophy, irregular hepatocyte cells, cytoplasmic vacuole expansion, nuclear displacement and disappearance, and inflammatory cell infiltration. These results are consistent with earlier research on various terrestrial species (Hall et al. 2000; Liu et al. 2018) and fish (Liu et al. 2016; Li et al. 2019a, 2019b; Khieokhajonkhet et al. 2022).

In respect to the kidney tissues, in the present study, exposure to 31 °C for 28 days resulted in hyperemia, extensive glomerular congestion, and tubular necrosis. This is consistent with previous studies that have shown the damaging effects of high temperature on renal tissues in fish. For example, Hernández-López et al. (2018) observed glomeruli and tubular necrosis, scattered cells in melanomacrophage centers, and disrupted tissue organization in Pacific sardine (*Sardinops sagax caeruleus*) exposed to high temperature (23 to 30 °C) for 25 days. Similarly, Jing et al. (2023) found that Siberian sturgeon (*Acipenser baerii*) exposed to different water temperatures (20 °C, 24 °C, and 28 °C) for 8 days showed heavy lymphocyte infiltration in the kidney tissues.

## Conclusions

The results of this study indicated that the optimum rearing temperature for European seabass (*D. labrax* L.) was 23–26 °C. The fish performed better at both water temperatures compared to other water temperatures. This is supported by the fact that there were no significant differences in growth and biochemical indices at those temperatures. It should be noted that rearing fish at higher temperatures (31 °C) resulted in high levels of stress and increased expression of oxidative/antioxidative genes (*SOD* and *CAT*). Additionally, histopathological examination of gill, liver, and kidney tissues from fish reared at higher temperatures (31 °C) showed significant damage, leading to a decline in fish performance and health status. Further research is needed to investigate the adaptation strategies to enhance fish performance under thermal stress conditions.

## Data Availability

No datasets were generated or analysed during the current study.
